# Fabrication of microstructured binary polymer brush “corrals” with integral pH sensing for studies of proton transport in model membrane systems†
†Electronic supplementary information (ESI) available: Experimental section, together with additional experimental data including the evolution of dry brush ellipsometric thickness *versus* time of OEGMA grown from silicon wafers functionalized with CMPTS followed by irradiation and reaction with glycerol and 2-bromoisobutyryl bromide; XPS measurements for silicon wafers functionalized with CMPTS followed by irradiation and reaction with glycerol and 2-bromoisobutyryl bromide and fluorescence spectra and confocal laser scanning images of NBC-labelled PCysMA brushes excited at 488 nm. See DOI: 10.1039/c7sc04424k


**DOI:** 10.1039/c7sc04424k

**Published:** 2018-01-15

**Authors:** J. Madsen, R. E. Ducker, O. Al Jaf, M. L. Cartron, A. M. Alswieleh, C. H. Smith, C. N. Hunter, S. P. Armes, G. J. Leggett

**Affiliations:** a Department of Chemistry , University of Sheffield , Brook Hill , Sheffield S3 7HF , UK . Email: peterjeppemadsen@googlemail.com ; Email: s.p.armes@sheffield.ac.uk ; Email: Graham.Leggett@sheffield.ac.uk; b Department of Molecular Biology and Biotechnology , University of Sheffield , Western Bank , Sheffield S10 2TN , UK; c King Saud University , Riyadh , Saudi Arabia; d Krebs Institute for Mechanistic Biology , University of Sheffield , Sheffield S10 2TN , UK

## Abstract

Binary brush structures consisting of poly(cysteine methacrylate) (PCysMA) “corrals” enclosed within poly(oligoethylene glycol methyl ether methacrylate) (POEGMA) “walls” are fabricated simply and efficiently using a two-step photochemical process. First, the C–Cl bonds of 4-(chloromethyl)phenylsilane monolayers are selectively converted into carboxylic acid groups by patterned exposure to UV light through a mask and POEGMA is grown from unmodified chlorinated regions by surface-initiated atom-transfer radical polymerisation (ATRP). Incorporation of a ratiometric fluorescent pH indicator, Nile Blue 2-(methacryloyloxy)ethyl carbamate (NBC), into the polymer brushes facilitates assessment of local changes in pH using a confocal laser scanning microscope with spectral resolution capability. Moreover, the dye label acts as a radical spin trap, enabling removal of halogen end-groups from the brushes *via in situ* dye addition during the polymerisation process. Second, an initiator is attached to the carboxylic acid-functionalised regions formed by UV photolysis in the patterning step, enabling growth of PCysMA brushes by ATRP. Transfer of the system to THF, a poor solvent for PCysMA, causes collapse of the PCysMA brushes. At the interface between the collapsed brush and solvent, selective derivatisation of amine groups is achieved by reaction with excess glutaraldehyde, facilitating attachment of aminobutyl(nitrile triacetic acid) (NTA). The PCysMA brush collapse is reversed on transfer to water, leaving it fully expanded but only functionalized at the brush–water interface. Following complexation of NTA with Ni^2+^, attachment of histidine-tagged proteorhodopsin and lipid deposition, light-activated transport of protons into the brush structure is demonstrated by measuring the ratiometric response of NBC in the POEGMA walls.

## Abstract 2

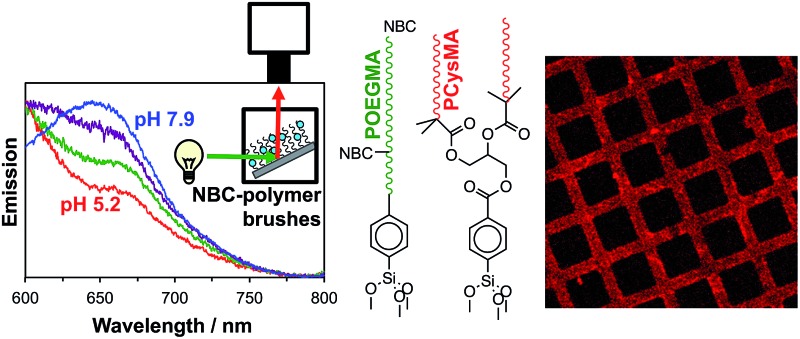
Binary polymer brush microstructures incorporating ratiometric fluorescent pH indicators enable *in situ* studies of light-activated transmembrane proton transport by proteorhodopsin.

## Introduction

The ability to detect local changes in pH is important in many areas of science, for example in the detection of disease;^1^–^6^ in bioreactors where changes in pH may be correlated with changes in microorganism performance;^7^–^10^ and in blood concentrates and cell growth media where the presence of metabolites resulting from bacterial infection may lead to a change in pH.^11^,^12^ The development of methods for rapid, non-invasive assessment of changes in pH in complex media is thus an important area of research.

The proton-motive force plays a central role in biology. For example, in photosynthetic systems, the maintenance of a transmembrane proton gradient is necessary to drive the conversion of ADP to ATP by ATPsynthase.^13^ The development of experimental platforms that enable *in situ* characterization of localised pH changes due to the action of membrane proteins is therefore highly desirable. Supported lipid bilayer systems (SLBs) are attractive models for membrane systems, offering the potential for detailed physicochemical investigation of phenomena which otherwise occur in microscopic systems (cells, organelles) that are more difficult to examine.^14^–^17^ However, an important obstacle to progress in this field has been the lack of methods for lifting SLBs sufficiently far above a solid substratum to facilitate the inclusion of functional transmembrane proteins. Recently we demonstrated that poly(cysteine methacrylate) (PCysMA) brushes formed by surface initiated atom-transfer radical polymerisation (ATRP) provide a cushion for SLBs that enables high lipid mobilities to be maintained.^18^ To make measurements of transmembrane proton transport in such membranes, a microsystem is required in which membrane components are confined spatially, protein function is maintained and where there is the capacity for local pH measurement. The present work describes the design of a binary polymer brush structure that achieves these characteristics.

Fluorescence-based techniques for measuring changes in pH are attractive because they have high sensitivity, enabling detection in small volumes.^19^–^21^ A wide range of fluorescence-based pH probes have been used for measuring the local pH in complex media.^19^,^20^,^22^,^23^ In ratiometric sensors, the pH change is detected by a shift in the emission spectrum rather than by a change in the fluorescence intensity. Compared to probes based solely on emission intensity, such probes are less susceptible to problems arising from non-specific quenching, photobleaching, variations in the intensity of the light source and background scattering.^19^

Polymer brushes provide an attractive platform for fluorescent sensors. The statistical incorporation of a comonomer dye into a polymer backbone suppresses dye–dye interactions,^24^ which allows higher dye concentrations to be used while retaining a linear output. This may enhance both the dynamic range and sensitivity. The use of fouling-resistant brushes prevents biofilm formation^25^ and impedes diffusion of biomacromolecules while allowing small molecules to diffuse rapidly through the brush layer.^26^ There appears to be remarkably few examples of fluorescent brush sensors in the literature. Yang *et al.* used the electrostatic attraction between poly(acrylic acid brushes) and a fluorescent ammonium tetraphenylethylene dye to measure the concentration of 2,4,6-trinitrotoluene,^27^ while Liu's group prepared an elegant FRET-based fluorescent thermometer from silica nanoparticles grafted with layers of thermoresponsive poly(*N*-isopropylacrylamide) (PNIPAM) brushes.^28^ Nese *et al.* used multifunctional macroinitiators to prepare fluorescein-containing pH-responsive ‘bottle brush’ copolymers that were non-fluorescent below pH 7 but fluorescent above.^29^ In addition, fluorescently-labelled brushes have been used to assess brush detachment from surfaces.^30^,^31^

A number of workers have explored the fabrication of multicomponent polymer brushes. Konradi and Rühe prepared two-component brushes from azo initiator-functionalized substrates by photopolymerisation of one monomer through a mask, followed by thermal polymerisation of a second monomer from non-irradiated initiator groups.^32^ Zhou *et al.* constructed micron-sized two-component brush structures on gold *via* photolithographic removal of a non-patterned polymer brush followed by initiator functionalization and polymerisation from the exposed areas.^33^ Huck's group have prepared multicomponent polymer brushes using sequential microcontact printing of a thiol-functional ATRP initiator and gold^34^ and of polydopamine-based initiators on passivated polymer brushes, followed by brush growth.^35^ Capillary force lithography has been exploited to pattern a polystyrene thin film deposited over a preformed ATRP initiator layer. Following brush growth from the exposed initiator pattern, the residual polystyrene was removed and a second brush was grown.^36^ The preparation of two-component brushes using complementary polymerisation techniques for brush growth has also been reported.^37^,^38^ For example, patterned initiators or chain transfer agents can be attached using masks. Yom *et al.* reported the preparation of three-dimensional multicomponent brushes on silicon or glass substrates by applying a photoresist to a non-patterned brush grown using ATRP. This was followed by resist removal using a mask and brush growth from exposed areas.^39^ Recently, Chapman *et al.* used photodeprotection chemistry in conjunction with ATRP to prepare three-dimensional two-component brushes.^40^ A similar approach was used by Johnson *et al.* to prepare two-component brushes in which one type of brush was capable of supporting lipid bilayers.^41^ In addition, the preparation of complex brush structures using Iridium-catalysed, light-mediated polymerisation has been reported.^42^

Here we describe a simple approach to the formation of binary polymer brushes, based around photochemical patterning of a chlorinated monolayer. We demonstrate the fabrication of structures consisting of corrals of proteins supported on PCysMA, surrounded by poly(oligoethylene glycol methyl ether methacrylate) (POEGMA) walls that contain a ratiometric fluorescent pH indicator, Nile Blue 2-(methacryloyloxy)ethyl carbamate (NBC), for measurement of local pH. Selective functionalization of PCysMA facilitates site-specific attachment of histidine-tagged proteorhodopsin. After deposition of lipids, light-activated transport of protons into the brush structure is demonstrated by measuring the ratiometric response of NBC in the POEGMA walls.

## Results and discussion

**Scheme 1:**
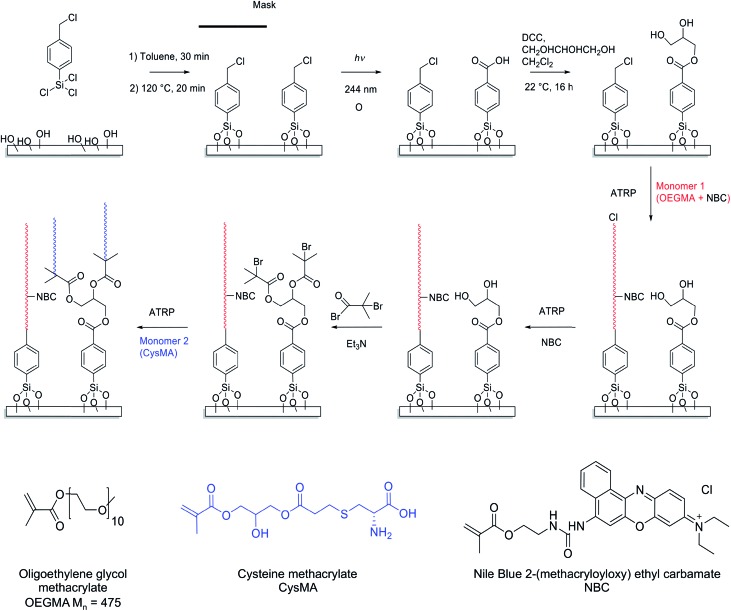
Formation of two-component brush patterns. After rigorous cleaning, surface silanols were functionalised with 4-(chloromethyl)phenyltrichlorosilane (CMPTS). A lithographic mask was placed over the CMPTS-patterned surfaces and UV irradiation was conducted in air using a total dose of 5 J cm^–2^, which oxidises the benzyl chloride groups to produce benzoic acid. The resulting carboxylic acid groups were esterified using excess glycerol in the presence of dicyclohexylcarbodiimide, DCC. The remaining benzyl chloride groups were used to initiate ATRP of OEGMA. When the desired POEGMA brush thickness was achieved, the growing brush chains were deactivated by adding one equivalent of NBC to CuBr to the reaction mixture. The hydroxyl groups in the irradiated areas were then functionalised with 2-bromoisobutyryl bromide and the resulting 2-bromoester groups were utilized to initiate the surface ATRP of CysMA to form well-defined two-component brush patterns comprising POEGMA and PCysMA.

Scheme 1 depicts the strategy developed to facilitate the preparation of two-component polymer brush structures. A planar surface (either glass or silicon with a native oxide layer) is first functionalized using 4-(chloromethyl)phenyl trichlorosilane (CMPTS). The terminal Cl of this adsorbate acts as an initiator for ATRP in Scheme 1, without any modification.^43^ Irradiation of the chloromethylphenyl groups with UV light in the presence of air leads to C–Cl bond cleavage followed by aerial oxidation to yield an aldehyde and, at high UV doses, further photo-oxidation to a carboxylic acid. Careful control of the irradiation dose determines whether the resulting surfaces are predominantly coated with aldehyde- or carboxylic acid groups.^44^,^45^ Neither of these two groups can act as an ATRP initiator, hence no polymer brush growth is observed in the irradiated areas. In the present study, conditions were chosen to ensure that the surfaces comprised predominantly carboxylic acid groups.^51^ This species was derivatised in two steps: carbodiimide coupling chemistry was utilized in the presence of excess glycerine to give an initial hydroxy-functional surface, with subsequent esterification using 2-bromoisobutyryl bromide in the gas phase producing the desired 2-bromoester ATRP initiator sites (see Scheme 1).

### Preparation of fluorescently labelled and unlabelled PCysMA and POEGMA brushes

**Fig. 1:**
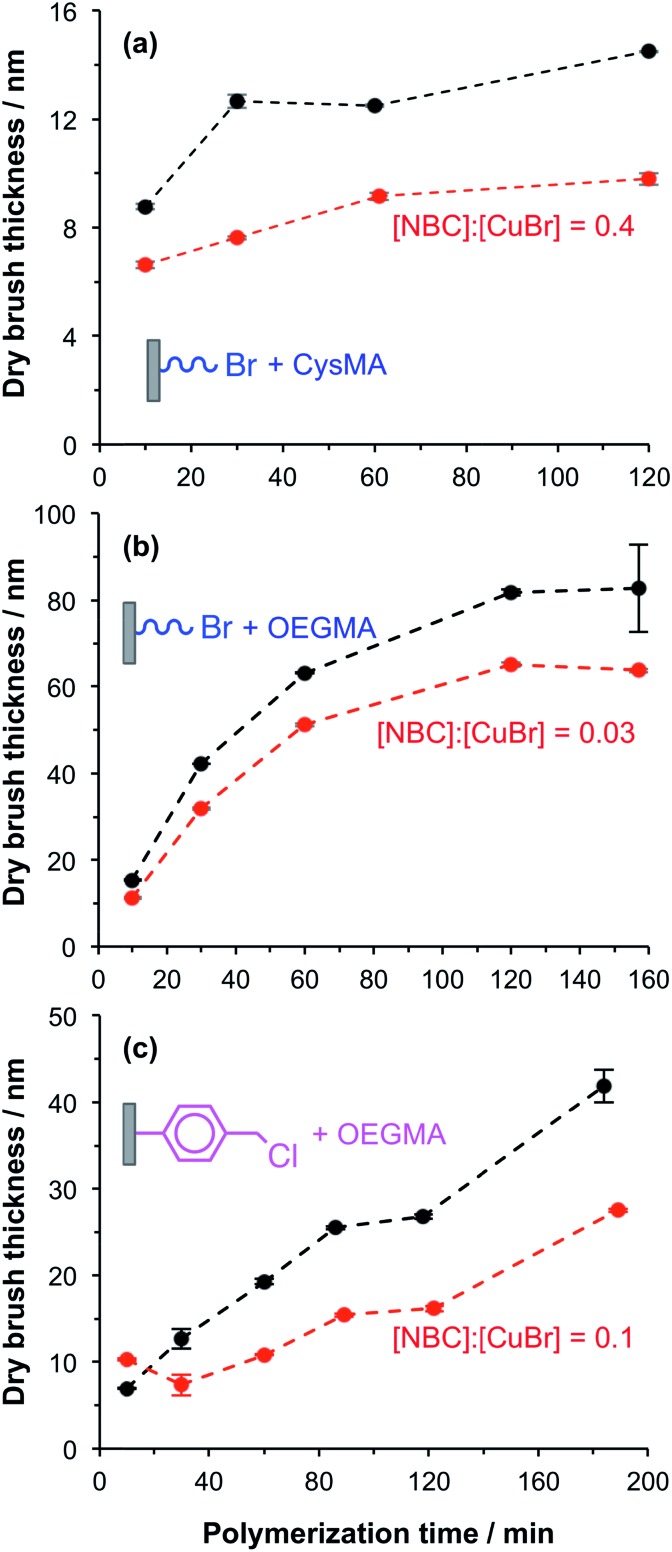
Effect of adding Nile Blue 2-(methacryloyloxy)ethyl carbamate (NBC) on the kinetics of surface ATRP of CysMA and OEGMA from planar substrates. (a–c) Show the evolution of the ellipsometric dry brush thickness with polymerisation time for the growth of PCysMA and POEGMA brushes from initiator-functionalized planar silicon wafers at 30 °C. Black dots represent brush syntheses conducted with no NBC in the reaction mixture and red dots represent experiments in which NBC was present. (a) APTES–BiB functionalized wafers; [CysMA] = 0.88 mol kg^–1^ [CysMA] : [bpy] : [Cu(i)Br] : [Cu(ii)Br_2_] : ([NBC]) = 70 : 3 : 1 : 0.5 : (0.4). (b) APTES–BiB functionalized wafers; [OEGMA] (DP ∼ 8) = 1.02 mol kg^–1^ [OEGMA] : [bpy] : [Cu(i)Br] : [Cu(ii)Br_2_] : ([NBC]) = 62 : 3 : 1 : 0.3 : (0.03). (c) CMPTS functionalized wafers; [OEGMA] = 1.01 mol kg^–1^, [OEGMA] : [CuBr] : [Cu(ii)Br_2_] : [Bipy] : ([NBC]) molar ratio = 60 : 1.0 : 0.3 : 2.5, (0.1), in H_2_O. In (b) and (c), *M*_n_ = 475 g mol^–1^.

In a control experiment, silicon and glass planar surfaces were first functionalized using (3-aminopropyl)triethoxysilane (APTES),^46^ followed by amidation of the primary amine surface groups with 2-bromoisobutyryl bromide (BiB) to yield ATRP initiator sites (APTES–BiB). This synthetic strategy is widely used for surface ATRP studies and is known to yield dense brush layers. Unpatterned fluorescently labelled PCysMA^47^ and POEGMA brushes were prepared by surface ATRP in the presence and absence of NBC.^48^ NBC is a methacrylic monomer and the labelled brushes are thus statistical copolymers. The fraction of dye-labelled repeat units in the brush is controlled *via* the relative amount of NBC in the reaction mixture. The dry brush thickness was monitored as a function of time using ellipsometry. In general, the presence of 0.03 to 0.40 equivalents of NBC relative to copper(i) retarded the rate of surface polymerisation and hence reduced the final mean brush thickness (see Fig. 1a and b). Such behaviour was expected because phenoxazine dyes in general, and nile blue in particular, have been reported to act as chain transfer agents.^48^,^49^

The aromatic phenoxazine group in NBC is separated from the polymerisable methacrylate group by two carbon atoms. In principle, this species may act either as a monomer or as a chain transfer agent. Previous work has shown that NBC can be used to label copolymer chains during their ATRP synthesis, while retarding the overall rate of polymerisation. This suggests that it may simultaneously act both as a monomer and a chain transfer agent, as shown in the ESI (Scheme S1†).

POEGMA brushes were grown from CMPTS-functionalized surfaces in the presence of 0.1 equivalents NBC relative to copper(i). The mean dry brush thickness exhibited a linear increase as a function of reaction time, with a growth rate of approximately 10 nm h^–1^ as judged by *ex situ* ellipsometry (see Fig. 1c). Such behaviour suggests well-controlled brush growth.^50^ In the absence of NBC, the rate of brush thickness growth was approximately 20 nm h^–1^ from CMPTS but some non-linearity was observed at longer polymerisation times (see Fig. 1c). Although this rate is twice that observed in the presence of the NBC quencher, it is nevertheless significantly lower than that observed for POEGMA growth from APTES–BiB surfaces (see Fig. 1b and c). This is probably related to the subtle difference in chemical structure between the initiator sites.^51^ Despite the slower rate of polymerisation, dye-labelled POEGMA brushes could be grown reproducibly with mean dry brush thicknesses of 20–30 nm from CMPTS-functionalized planar surfaces.

The growth of PCysMA brushes from CMPTS-functionalized surfaces that had been irradiated and converted into ATRP initiator sites (see step 3 in Scheme 1) proceeded at comparable polymerisation kinetics to those reported from APTES–BiB surfaces (see Fig. S1†).^47^ This result is somewhat surprising since APTES–BiB is an amide-based ATRP initiator, which are generally considered to be less efficient than ester-based ATRP initiators.^52^ In addition, full conversion of acid groups into initiator sites according to the pathway shown in Scheme 1 should give two initiator groups per irradiated CMPTS group; all other things being equal, the mean brush thickness should increase with the density of initiator groups. The surface composition of the CMPTS surface layer after its oxidation and functionalization was thus characterized by XPS. The Br/C atomic ratio for the irradiated CMPTS initiator was 0.04 (theoretical value = 0.11), compared to that of 0.08 for the APTES–BiB initiator (theoretical value = 0.14). Thus the XPS data suggest an overall conversion for the three-step synthesis of irradiated CMPTS initiator from CMPTS of *ca.* 40% based on the Br/C atomic ratio. This should be compared to an overall conversion of 60% based on the Br/C ratio determined for the APTES–BiB initiator. Such surface reactions are often incomplete because of the sterically confined environment.^53^ Moreover, the CMPTS initiator was prepared in three steps. The photochemical conversion of CMPTS to carboxylic acid has been reported to be around 85% based on XPS data under the conditions utilized herein, with the remaining 15% corresponding to be non-chlorine containing species.^51^

No further optimization of the initiator formation and brush growth from the irradiated CMPTS was conducted since it was possible to grow PCysMA brushes of up to 20 nm dry brush thickness from the as-prepared surfaces (see Fig. S1†).

### Preparation of binary brush patterns

The strategy to prepare binary brush patterns is shown in Scheme 1. First, CMPTS-functionalized silicon or glass substrates were patterned by UV irradiation using a photomask. In order to suppress undesired interactions between the carboxylic acid groups and the basic ligands used for the ATRP catalyst, the resulting carboxylic acid patterns were esterified with excess glycerol using carbodiimide coupling chemistry to afford hydroxy-functionalized patterns prior to polymerisation of OEGMA (see Scheme 1).

POEGMA brushes were prepared by ATRP of OEGMA in the presence of NBC (NBC/CuBr molar ratio = 0.10) at 30 °C, with the intact Cl atoms that were masked during UV exposure acting as the initiator groups. After growth of this first patterned brush, it was necessary to terminate the living ends of the surface-grafted polymer chains. The feasibility of using NBC to achieve this, by polymer radical transfer to the dye label was examined. This strategy may present a convenient alternative to the capping strategies based on substitution of bromine with azide^33^,^34^ or dehalogenation using hydrides that are reported in the literature. After polymerisation of OEGMA for 1 h, additional NBC (one equivalent relative to Cu(i)Br) was added as a degassed ethanolic solution directly to the polymerisation mixture. The reaction mixture was then left for a further 12 h at 30 °C.

**Fig. 2:**
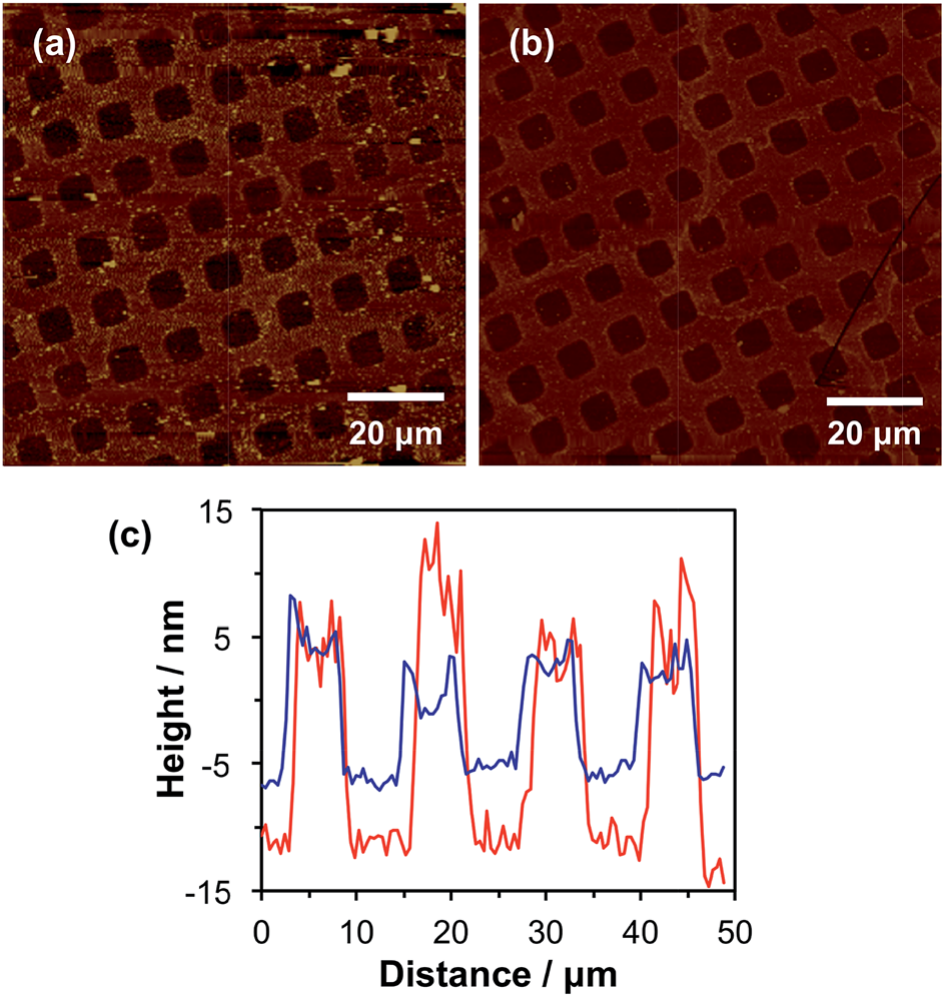
(a) AFM tapping mode height image of a microstructured POEGMA brush, grown from chlorine-terminated regions of a photopatterned CMPTS layer (bars). The surface-grafted POEGMA chains are capped with NBC and the irradiated areas then functionalized using 2-bromoisobutyryl bromide. (b) Tapping mode height image of a binary brush pattern formed by ATRP of CysMA from the Br-functionalized regions (squares) of the patterned structure shown in (a) to form patterned PCysMA brushes. (c) Section through the images shown in (a) (red line) and (b) (blue line).

This protocol efficiently terminated the brush chain-ends, as no further brush growth was observed as demonstrated in the tapping mode AFM images shown in Fig. 2a and b. Fig. 2a shows a tapping mode AFM image of micropatterned POEGMA brushes capped with NBC followed by esterification of the hydroxy groups in the wells using excess 2-bromoisobutyryl bromide. The corresponding height profile (see Fig. 2b, red line) indicates that the POEGMA brushes have a mean dry brush height of approximately 20 nm, which is consistent with the corresponding ellipsometry data obtained for non-patterned POEGMA brushes after 1 h growth (see Fig. 1c).

Binary brush patterns were prepared by polymerizing CysMA from the 2-bromoisobutyrate in the wells (Fig. 2c). In this case, the CysMA/Cu(i)Br molar ratio was 30, which is reported to give a dry brush thickness of 6–8 nm within 2 h as judged by ellipsometry.^47^ The difference in brush height before and after CysMA polymerisation as determined by AFM is approximately 6 nm, which is close to the expected value (see Fig. 2c, blue line).

**Fig. 3:**
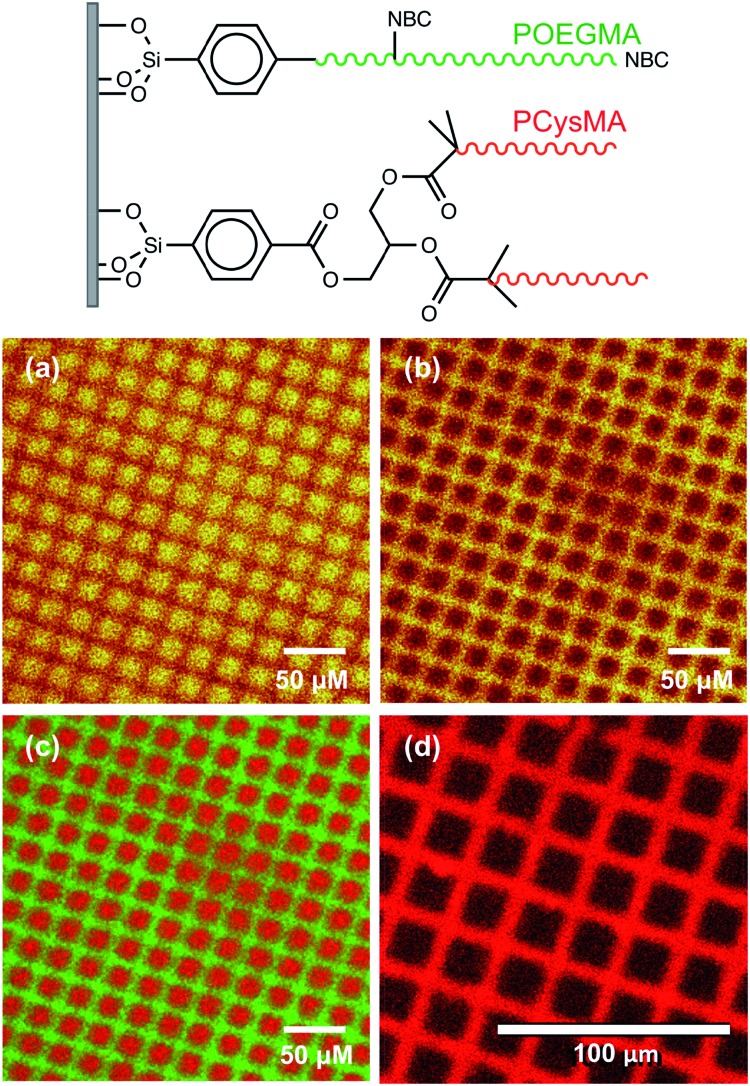
ToF-SIMS images recorded for patterned polymer brushes formed using a 1000 mesh grid as a mask. NBC was present throughout the surface polymerisation of OEGMA so it can be statistically incorporated within the POEGMA brush as a comonomer and also present at the chain terminus as a capping agent. (a) Image formed by mapping summed intensities of ions assigned to PCysMA (CN^–^, S^–^, HS^–^ and C_2_H_5_S_2_^–^). (b) Image formed by mapping summed intensities of ions assigned primarily to POEGMA (C_2_H_3_O^–^, C_2_H_2_O_2_^–^, C_2_H_5_O_2_^–^). (c) Overlay of ions assigned to PCysMA (red) and POEGMA (green). (d) Confocal laser scanning image of this surface patterned brush layer immersed in water at pH 7.9, exhibiting fluorescence from regions masked during the initial UV exposure (bars). Excitation laser wavelength is 543 nm, while the emission wavelength is 600–700 nm.

POEGMA-PCysMA binary brush patterns were formed using a 1000 mesh TEM grid as a mask and characterized using imaging ToF-SIMS (Fig. 3). Fig. 3a shows an image formed by mapping the summed intensities of the sulfur- and nitrogen-containing ions, which are assigned to PCysMA. Bright contrast is observed in the square regions, which were exposed to UV irradiation. Fig. 3b shows the summed intensities of the most prominent ions observed in the SIMS spectrum of POEGMA. A strong signal is observed in the masked regions (bars) as expected. The absence of any ions that are characteristic of PCysMA in the masked regions suggests efficient removal of terminal halogen atoms from the POEGMA brush chains. This important point is confirmed by overlaying the intensities of ions that are characteristic of PCysMA and POEGMA in Fig. 3c. Finally, confocal fluorescence microscopy studies were conducted on this binary brush substrate (Fig. 3d). Bright contrast was observed in regions that had been masked during UV exposure. POEGMA brushes incorporating NBC were grown from these regions, and the presence of this dye label ensures that these regions exhibit bright contrast in fluorescence images. The pattern of bright bars in Fig. 3d corresponds to the bars that were masked during UV exposure, as expected. The strong contrast here suggests that dehalogenation was quantitative.

Efficient chain capping using NBC was achieved simply by adding this dye to the ATRP reaction solution *in situ.* This strategy was preferred to other capping methods because it allowed simultaneous chain termination and introduction of a pH-responsive fluorescent dye.

### pH-Dependent fluorescence of NBC-labelled brushes

**Fig. 4:**
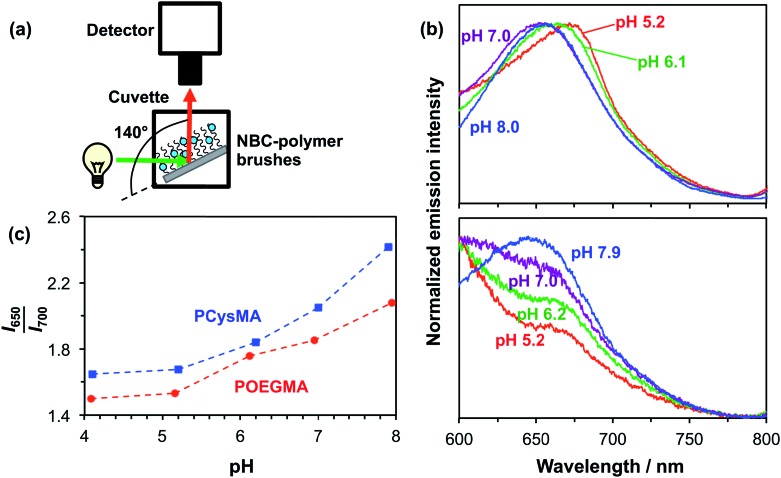
Spectroscopic studies of the pH-dependent fluorescence of NBC-labelled PCysMA brushes and NBC-labelled POEGMA brushes on glass slides using a spectrofluorimeter. (a) Schematic experimental set-up. A brush-functionalized glass slide was placed in a cuvette at an angle of ∼140° to the excitation beam. The cuvette was filled with 0.1 M phosphate buffer adjusted to the desired pH and emission spectra were recorded at an excitation wavelength of 543 nm. (b) Top: emission spectra obtained for ∼20 nm POEGMA brushes ([OEGMA]/[NBC] = 600) at pH 5.2–8.0. Bottom: emission spectra obtained for ∼10 nm PCysMA brushes ([PCysMA]/[NBC] = 460). (c) Dependence of the *I*_650_/*I*_700_ intensity ratio for PCysMA and POEGMA as a function of solution pH. Spectrophotometer settings: excitation slit = 10 nm, emission slit = 20 nm. Scan rate – 1200 nm min^–1^. PMT = 950 V.

Initially, the pH-dependent fluorescence of labelled brushes was recorded using a spectrofluorimeter. A glass slide coated with unpatterned PCysMA or POEGMA brushes labelled with NBC was placed in a fluorescence cuvette, as shown in Fig. 4a.^54^ This cuvette was then filled with a 0.1 M phosphate buffer at the desired pH. This set-up enabled a qualitative assessment of the substrate fluorescence spectra on varying the excitation wavelength. Fig. 4b shows the normalized fluorescence spectra obtained for a glass slide with NBC-labelled POEGMA on increasing the pH from 5.2 to 7.9. This pH variation leads to a hypsochromic shift in the emission spectrum, such that *λ*_max_ is reduced from 675 nm at pH 5.2 to 650 nm at pH 7.9. These observations correlate well with previous studies of the pH-dependence of the NBC-dye incorporated into water-soluble polymers in solution.^48^ A similar pH-dependence can be observed for NBC incorporated into PCysMA brushes in Fig. 4b, although the signal-to-noise ratio is weaker in this case because these brushes were only half as thick as the POEGMA brushes. The apparent increase in the fluorescence intensity observed at short wavelengths is because there is an NBC emission band at 585 nm that appears to be sensitive to its local environment (see Fig. S2 in the ESI† for spectra recorded over a wider wavelength range). This 585 nm band is relatively less prominent for POEGMA, presumably because this neutral brush provides a significantly different local environment to that conferred by the zwitterionic PCysMA brush.

Use of these dye-labelled brushes as ratiometric probes for monitoring changes in pH is demonstrated in Fig. 4c, where the *I*_650_/*I*_700_ intensity ratio is plotted as a function of pH for the two labelled brushes. This ratio increases between pH 5 and pH 8, which reflects the hypsochromic shift.

**Fig. 5:**
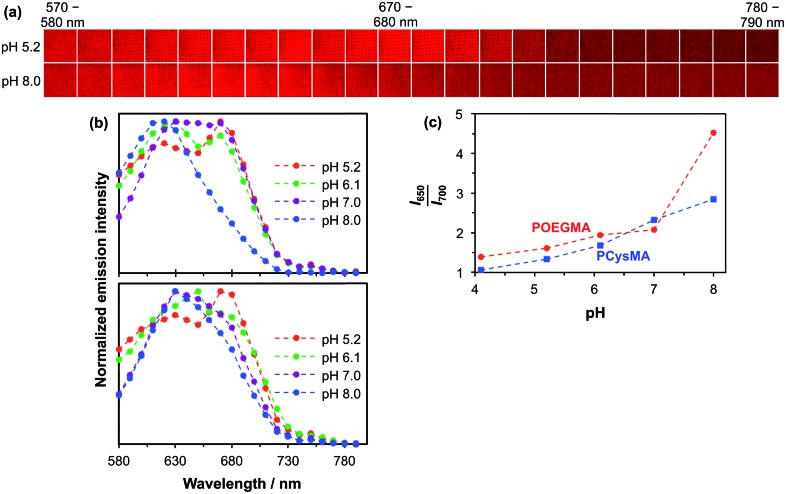
pH-Dependent fluorescence observed for NBC-labelled PCysMA and POEGMA brushes using a confocal laser scanning microscope (CLSM) with spectral resolution capability. (a) Microscope images excited at 543 nm with 10 nm resolution. (b) Top: pH-dependent fluorescence spectra based on CLSM images of NBC-labelled POEGMA brushes (approximate dry brush thickness = 60 nm). Bottom: pH-dependent fluorescence spectra based on CLSM images of NBC-labelled PCysMA brushes (approximate dry brush thickness = 10 nm). (c) Ratiometric fluorescence *vs.* pH plot obtained for these PCysMA and POEGMA brushes.

The detection of pH-induced fluorescence changes in micron-sized patterns was accomplished using a confocal laser scanning microscope with spectral imaging capability (CLSM-SI). Fig. 5a shows the resulting fluorescence microscope images obtained for a 2000 mesh pattern of a NBC-labelled POEGMA brush, which is excited using a green laser at 543 nm. The images are recorded at 10 nm intervals between 570 nm and 790 nm at pH 5.2 and 8.0, respectively. The shift in the maximum emission at these two pH values can be observed directly as the low-pH images are more intense at longer wavelengths than the high-pH images. The resulting plot of the normalized intensity of the fluorescently-labelled POEGMA brush patterns against wavelength is shown in Fig. 5b. The corresponding plot for NBC-labelled PCysMA brushes is shown in Fig. 5b. These plots are consistent with the fluorescence spectra recorded using a spectrofluorimeter (*e.g.*, compare with Fig. 4b for POEGMA brushes and Fig. 4b for PCysMA brushes) despite the lower spectral resolution of the CLSM set-up. Importantly, the fluorescence emission is characterized by a hypsochromic shift at higher pH similar to that observed using the spectrofluorimeter. In addition, the plot of the *I*_650–660_/*I*_700–710_ fluorescence intensity ratio exhibits the same general trend as the *I*_650_/*I*_700_ intensity ratio obtained using the spectrofluorimeter, see Fig. 5c, confirming that the pH-sensitivity of the patterned POEGMA brushes is not significantly different from that of the unpatterned brushes.

Although the spectroscopic observations made using the spectrofluorimeter and CLSM-SI are certainly comparable, the respective curves are not actually identical (compare Fig. 4c and 5c). This is most likely owing to differences in the wavelength response of the light detector. In addition, the wavelength ranges are different, which reflects the smaller measuring areas used in the microscope set-up. Thus the calibration curve used to monitor transmembrane proton transport (see below) should be the one shown in Fig. 5c, so that data are compared for spectra acquired using the same instrument.

### Site-specific attachment of proteins

Both POEGMA and PCysMA brushes are known to exhibit excellent antibiofouling properties.^47^,^55^ However, while the polyether side-chains in POEGMA are chemically inert, PCysMA comprises a primary amine and a carboxylic acid group in each monomer residue. In principle, both these functional groups can be modified selectively under mild conditions.^56^ In addition, we have recently reported that brushes can be derivatised solely at their near-surface simply by performing the desired reaction in a poor solvent for the brush, which leads to its collapse.^57^–^59^ This ensures that the majority of the PCysMA brush chain remains underivatised. Here we demonstrate selective, site-specific conjugation of a histidine-tagged transmembrane proton pump, proteorhodopsin, to the surface of PCysMA chains within PCysMA/POEGMA binary brush structures.

**Scheme 2:**
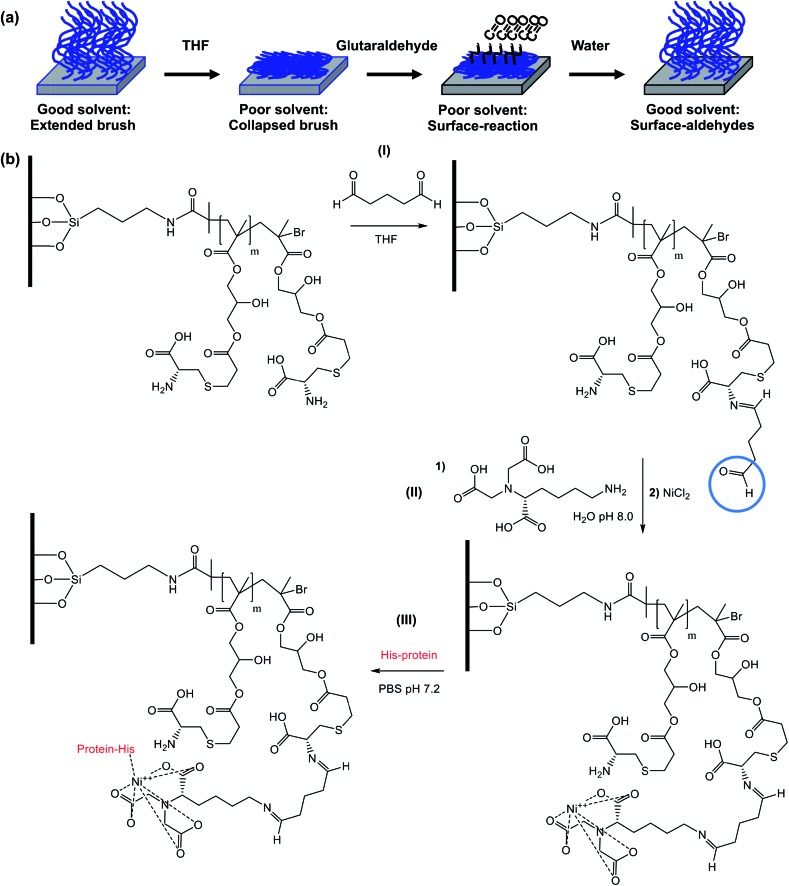
Selective functionalization of a PCysMA brush for covalent attachment of His-tagged proteins. (a) Schematic representation of the reaction of a PCysMA brush with excess glutaraldehyde in a poor solvent (THF). (b) Exploiting spatial functionalization with glutaraldehyde to produce protein-conjugated PCysMA brushes. (i) PCysMA brushes reacted with glutaraldehyde in THF for 20 h at 22 °C. (ii) First, reaction of aldehyde-functionalized PCysMA brushes with *N*_α_,*N*_α_-bis(carboxymethyl)-l-lysine hydrate (NTA) in water at pH 8.0 for 2 h at 22 °C to give NTA-functional brushes. Second, complexation of the NTA group with Ni^2+^ ions by exposure to a 1 M NiCl_2_ solution for 12 h at 22 °C. Third, attachment of His-tagged protein (green fluorescent protein, GFP or proteorhodopsin, pR) by incubation with 0.01 g dm^–3^ protein in PBS (pH 7.2) for 12 h at 4 °C. Brushes were washed and dried between each step. After the protein conjugation step, the protein-labelled brushes were kept hydrated by PBS and stored in the fridge at 4 °C prior to use in order to avoid denaturation of the protein.

The strategy for selective modification of PCysMA is shown in Scheme 2. Initially, its primary amine groups are reacted with excess glutaraldehyde in tetrahydrofuran, which is a non-solvent for this brush. In principle, glutaraldehyde can react with two amines to induce surface crosslinking between neighbouring brush chains. However, the large excess of this reagent combined with the poor solvent quality should ensure the formation of (near-)surface aldehyde groups.^60^

**Fig. 6:**
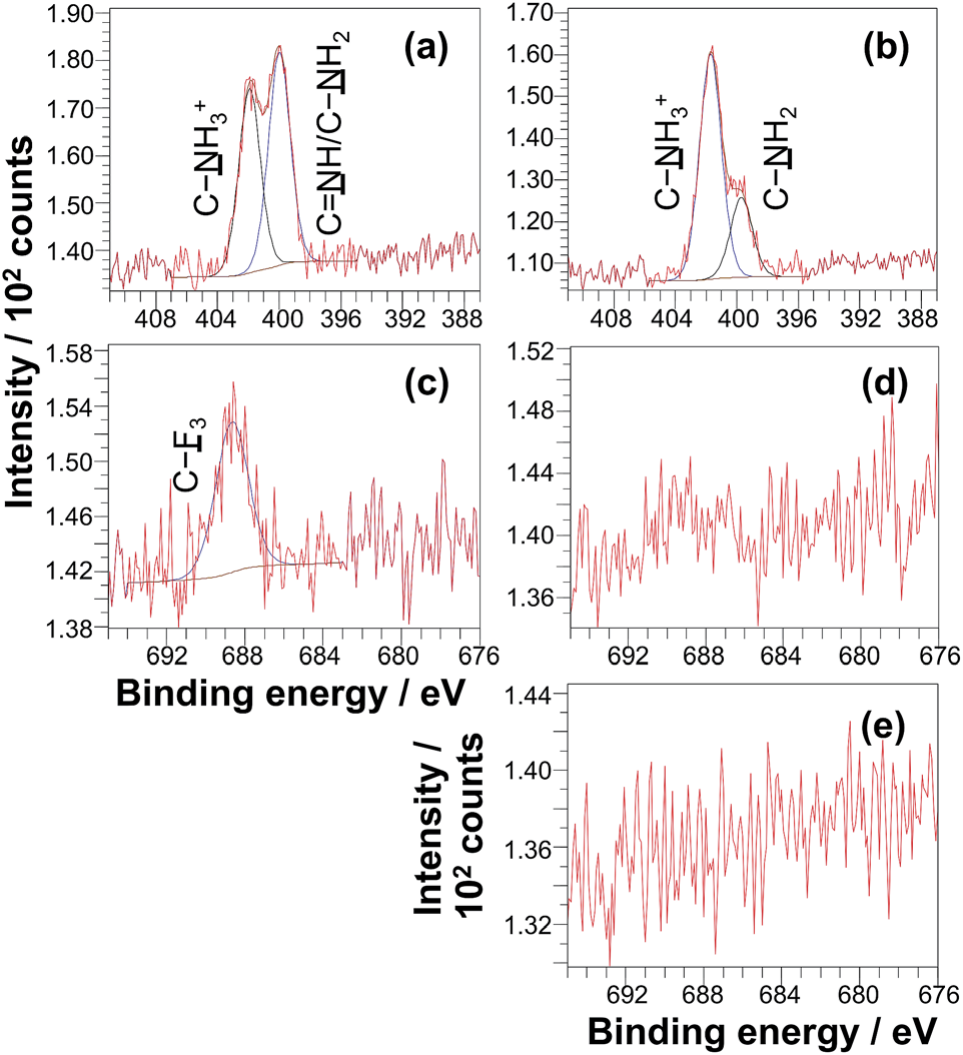
High resolution X-ray photoelectron spectra and sessile drop contact angles recorded for PCysMA brushes of approximately 10 nm dry brush thickness following surface chemical reactions. (a) N 1s spectrum of PCysMA after incubation with excess glutaraldehyde in THF. (b) N 1s spectrum of an as-prepared PCysMA brush. (c) F 1s spectrum recorded after incubation of a PCysMA brush with (i) excess glutaraldehyde in THF followed by (ii) exposure to gaseous trifluoroethylamine, TFEA, for 2.5 h at 22 °C. (d) F 1s spectrum of the same PCysMA brush after exposure to TFEA for 2.5 h at 22 °C but with no prior exposure to glutaraldehyde. (e) F 1s spectrum of an as-prepared PCysMA brush.

The surface modification reactions were confirmed by XPS. Fig. 6a shows the high resolution XPS N 1s spectrum recorded for a non-patterned PCysMA brush treated with excess glutaraldehyde in THF. This spectrum can be fitted with three components cantered at 401.5 eV, 400.2 eV and 399.3 eV, which correspond to C–NH_3_^+^, C=NH and C–NH_2_ respectively.^47^,^61^,^62^ The ratio of the area of the imine component to the sum of the areas of the amine and ammonium species is 0.28, suggesting that *ca.* 20% of the PCysMA residues reacted with aldehyde groups to form imines. Fig. 6a shows that the C–NH_3_^+^/C–NH_2_ atomic ratio is approximately unity after glutaraldehyde treatment. This is somewhat surprising, given that this ratio is around 8 : 2 prior to derivatisation with glutaraldehyde (see Fig. 6b).^47^ A possible explanation is that the presence of THF shifts the equilibrium between the zwitterionic and non-zwitterionic form of the pendent amino acid groups. Such a shift has been previously reported for simple amino acids when changing the solvent from water to ethanol, which has a somewhat higher dielectric constant compared to that of THF (24.5 *vs.* 7.6).^63^

To confirm the presence of surface aldehyde groups, the glutaraldehyde-treated PCysMA brushes were reacted with trifluoroethylamine (TFEA). Introduction of this fluorinated reagent yields a characteristic spectral signature that may be used to quantify the aldehyde content. Fig. 6c shows the high resolution F 1s XPS spectrum obtained after exposure of glutaraldehyde-treated PCysMA brushes to gaseous TFEA. An F 1s signal is clearly evident at 689 eV. Moreover, the water contact angle increased from 46.7° to 54.2°, which is consistent with covalent attachment of hydrophobic trifluoromethyl groups to the brush surface. In contrast, a control PCysMA brush that had not been treated with any glutaraldehyde but similarly exposed to TFEA (compare Fig. 6c and d) possesses a negligible F 1s signal. For PCysMA brushes reacted with glutaraldehyde prior to exposure to TFEA, the F/S atomic ratio was determined from the survey spectrum (not shown) to be *ca.* 0.10 given that there are three fluorine atoms per imine group, this suggests that less than 5% of the CysMA residues have been derivatised. Because around 20% of the CysMA residues had reacted with glutaraldehyde (based on the data shown in Fig. 6a, as discussed above), this indicates that less than a quarter of these derivatised repeat units subsequently react with TFEA.

**Fig. 7:**
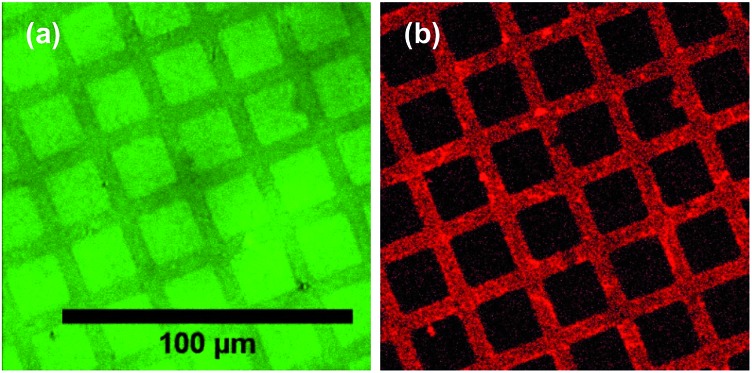
Attachment of His-tagged green fluorescent protein (GFP) to POEGMA-PCysMA binary brushes, where the PCysMA chains have been reacted with (i) glutaraldehyde, (ii) *N*_α_,*N*_α_-bis(carboxymethyl)-l-lysine hydrate, (iii) Ni(ii)Cl_2_ (see Experimental section for further details). (a) Excitation at 488 nm, emission at 500–600 nm. (b) Excitation at 543 nm, emission at 600–700 nm. The scale bar applies to both images.

A protein with a terminal oligohistidine sequence complexes strongly with surface-coordinated nickel ions, enabling site-specific binding to induce a uniform orientation at the interface.^64^–^66^ To conjugate proteins *via* this His-tag, surface aldehydes are reacted with the metal ligand *N*_α_,*N*_α_-bis(carboxymethyl)-l-lysine hydrate (NTA); after washing with water, nickel(ii) is attached to the brush chains *via* incubation with a Ni(ii)Cl_2_ solution^67^ and the brush is subsequently exposed to a solution of the relevant protein in PBS. Fig. 7 shows confocal laser scanning microscopy images obtained for a binary brush pattern consisting of PCysMA brush squares separated by bars of POEGMA brush prepared on a silicon wafer, after derivatisation with NTA/Ni^2+^ as described above and attachment of His-tagged green fluorescent protein (GFP). Excitation using a 488 nm laser reveals stronger fluorescence intensity between 500 nm and 600 nm within the PCysMA brush squares, which suggests selective labelling (see Fig. 7a). Weak fluorescence is also observed from the POEGMA bars. However, it should be noted that NBC does fluoresce weakly when excited at 488 nm (see Fig. S2†), which explains the background signal associated in the protein-resistant POEGMA regions. In contrast, laser excitation at 543 nm, which is a wavelength at which GFP does not absorb, yields fluorescence from only the NBC-derivatised POEGMA regions (see Fig. 7b).

### Reconstruction of a transmembrane proton transfer mechanism

**Fig. 8:**
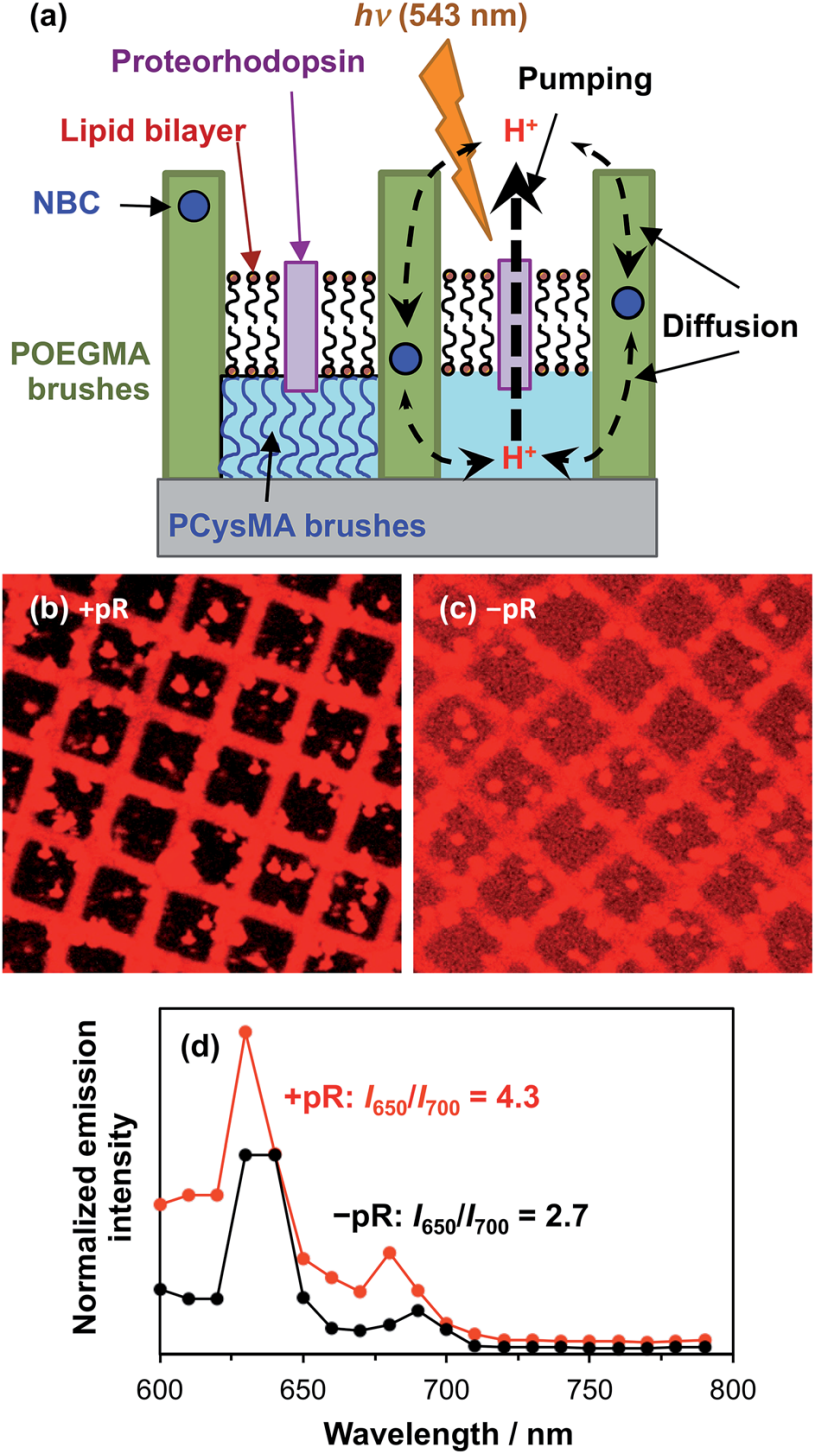
Proton pumping action of proteorhodopsin immobilized on a POEGMA-NBC/PCysMA binary brush structure. (a) Schematic of the set-up: pR is attached to the surface of the PCysMA brush using His-tag chemistry, which ensures that all pR are oriented in the same direction. Then a lipid bilayer is suspended on the PCysMA brush to form a lipid bilayer. Upon irradiation, pR pumps protons in a given direction through the lipid bilayer. The resulting pH difference is countered by diffusion through the POEGMA brush layers. However, since diffusion occurs more slowly than proton pumping, a net pH gradient is generated during irradiation. Diffusion ensures that equilibrium is achieved following irradiation. The time scale for reaching equilibrium was faster than what could be measured using the current setup. (b) Image formed by mapping the emission between 600 nm and 700 nm from POEGMA-NBC/PCysMA where pR is immobilized on PCysMA using a His-tag, following bilayer formation mediated by addition of a lipid vesicle suspension consisting of 1-palmitoyl-2-oleoyl-*sn*-glycero-3-phosphocholine doped with 0.5 mol% Texas Red® 1,2-dihexadecanoyl-*sn*-glycero-3-phosphoethanolamine, triethylammonium salt. (c) Emission between 600 nm and 700 nm for POEGMA-NBC/PCysMA without pR following *in situ* bilayer formation by addition of the lipid vesicle suspension. (d) Emission intensity from POEGMA-NBC walls for samples with and without immobilized pR. The *I*_650_/*I*_700_ emission intensity ratio is calculated in each case and inserted.

Proteorhodopsin is a membrane protein that is capable of pumping protons across a lipid membrane on irradiation with visible light.^68^ His-tagged proteorhodopsin (pR) was attached to PCysMA regions within POEGMA/PCysMA binary brush structures prepared on planar silicon wafers, with the POEGMA brush chains being labelled using the pH-responsive NBC dye (POEGMA-NBC/PCysMA). PCysMA brushes are known to support mobile lipid bilayers.^69^ Thus, reconstitution of PCysMA-conjugated proteorhodopsin with a lipid is expected to give a pR-functionalized lipid bilayer situated at the upper surface of this brush layer (see schematic shown in Fig. 8a).

Due to the oriented attachment of the light-sensitive protein, irradiation should lead to directional proton transport across the lipid bilayer. Back-diffusion of protons through the POEGMA brush, which are not isolated by a lipid bilayer, should lead to equilibrium becoming re-established when the light source is turned off. However, given that proton pumping is expected to be faster than the rate of diffusion, continuous illumination should lead to steady-state conditions in which the proton concentration is higher or lower on the brush side of the lipid bilayer relative to the bulk liquid. This should shift the emission of the NBC dye label, as described above.

Fig. 8b shows a confocal fluorescence image recorded for a POEGMA-NBC/PCysMA binary brush structure, where proteorhodopsin was first conjugated to the PCysMA brush (square regions) followed by reconstitution of the protein into a lipid bilayer *via in situ* fusion of lipid vesicles.^70^Fig. 8c shows the corresponding binary brush system without proteorhodopsin. In both cases residual lipid vesicles with a diameter of several microns can be observed. However, the difference in contrast between the brush walls and brush wells is significantly larger in the presence of pR than in its absence, when the low-level of background emission from the labelled lipids (present at ∼1% the concentration of NBC in the lipid-resistant POEGMA brushes,^36^,^68^ present as a diagnostic device to confirm membrane formation)^71^ appears to be more significant.

These data suggest a substantial modification occurs to the fluorescence emission from the NBC-labelled POEGMA “walls” relative to the levels in the PCysMA “corrals” that they enclose when pR is added to the corrals. The extent of this modification and its explanation may be analysed more quantitatively by exploiting the potential of NBC for ratiometric analysis of pH change (as demonstrated in Fig. 4 and 5). In particular, the *I*_650_/*I*_700_ emission ratio was measured for the two systems (Fig. 8d) and was found to be almost twice as high in the presence of pR than in its absence, indicating a substantial change in the pH in the POEGMA “walls”. Specifically, the local pH is lower in the absence of pR (compare Fig. 8d with Fig. 5c). This suggests that steady-state conditions due to active pumping have been achieved, as indicated in Fig. 8a.

Precise assessment of the pH directly from the *I*_650_/*I*_700_ emission ratio is complicated by the reflective properties of the underlying silicon wafer, leading to periodic ‘spikes’ in the emission spectra (at ∼630 and 680 nm) that are the result of constructive interference between direct fluorophore emission and the associated reflected emission.^72^ The positions of these spikes affect the *I*_650_/*I*_700_ emission ratio. In addition, the reflectivity of silicon is reduced at higher wavelengths,^73^ hence the reflected emission is attenuated at longer wavelengths compared to that for glass. Thus, it is likely that this emission ratio leads to an overestimate of the local pH. In general, polished surface-oxidised silicon wafers proved to be superior substrates compared to glass for preparing brushes and corrals. In principle absolute measurements of pH would make these structures even more useful but this would require the use of high quality glass. This potential refinement clearly merits further investigation.

We can nevertheless use these data to estimate the local pH in the POEGMA walls. An *I*_650_/*I*_700_ emission ratio of 4.3 in the presence of proteorhodopsin suggests a pH of around 8, whereas a *I*_650_/*I*_700_ ratio of 2.7 in the absence of proteorhodopsin suggests a pH of approximately 7.3 according to Fig. 5d. At the very least, the significant difference in emission ratio observed in the presence and absence of proteorhodopsin provides strong evidence for directional active proton pumping leading to the establishment of a changed local pH in the POEGMA “walls”.

As noted above, the proton-motive force plays a central role in biology. For example, in photosynthetic systems such as *Rhodobacter sphaeroides*, the maintenance of a transmembrane proton gradient is necessary to drive the conversion of ADP to ATP by ATPsynthase.^13^ The kinetics of ATP synthesis are determined by the composition and organisation of the membrane proteins in the chromatophore vesicle of *R. sphaeroides*.^74^,^75^ In the work described here, pR serves as a surrogate for several proteins that work in concert to sustain a transmembrane proton gradient during bacterial photosynthesis. Reconstruction of the membrane of the chromatophore vesicle in a corral such as that shown in Fig. 8 would enable the kinetics of transmembrane proton transport to be studied as a function of membrane composition, or would enable investigation of the effects of incorporation of modified membrane proteins. For example, reaction centre complexes were recently modified by attachment of yellow fluorescent protein to yield enhanced light energy capture in *R. sphaeroides*.^76^

The work described here is a proof-of-concept experiment, and future adaptations could be envisaged that might enable more complex processes to be explored. For example, an important unresolved question concerns the relationship between the pH developed in the polymer brush “corrals” described here and the pH that might develop on the interior of a vesicle. In the present case, proton transport into the PCysMA corrals is followed by diffusion of protons into the POEGMA walls. The diffusion coefficient for protons exiting the corrals is probably larger than that for proton transport through a vesicular membrane, meaning that the pH maintained in the corrals is different than would be the case for a vesicle of equivalent volume. A modification to the system described here would be to utilise solid walls (*e.g.* from microfabricated metal or oxide) and place the reporter inside the corrals, thus enabling the maintenance of the pH. In addition, the use of CLSM-SI allows spatial read-out of pH (in three dimensions), which in principle would allow mapping of pH-gradient at various points in the corrals. In principle this can be facilitated using the same dye, but it may be more convenient to use a combination of dyes with different spectral characteristics. Alternatively, a detailed kinetic analysis of the more open system described here should still yield the rate of transmembrane proton transport. Such studies are beyond the scope of the present work.

## Conclusions

Labelling of two hydrophilic polymer brushes, poly(oligoethylene glycol methacrylate) and poly(cysteine methacrylate) with a fluorescent dye (Nile blue 2-(methacryloyloxy)ethyl carbamate) has been demonstrated. The emission characteristics from these brushes vary systematically when switching the brush pH between pH 5 and pH 8. Specifically, the ratiometric response between emission intensities at 650 nm and 700 nm can be used to assess local changes in pH. This has been achieved using both a spectrofluorimeter and also a confocal laser scanning microscope with spectral imaging capability. The latter technique enables local changes in pH to be assessed for micrometre-sized brush patterns.

In addition, a novel method for the preparation of two-component double hydrophilic brushes on silicon and glass substrates has been developed. Substrates were first functionalized with chloromethylphenyltrichlorosilane. The immobilised benzyl chloride group can be used as an ATRP initiator, so it is possible to grow polymer brushes directly from such surfaces. UV irradiation conducted in air leads to *in situ* oxidation of the benzyl chloride groups to produce benzoic acid groups in high yield. The latter species cannot initiate polymerisation, but it can be conveniently converted to afford an initiator in two steps at a later stage. Thus, NBC-containing poly(oligoethylene glycol methacrylate) brushes were prepared first on a patterned CMPTS surface. Nile blue 2-(methacryloyloxy)ethyl carbamate proved to be a highly efficient *in situ* radical quencher that removed bromine in order to prevent further polymerisation from the POEGMA brush chains. Next, the carboxylic acid was converted into an ATRP initiator, which was used to grow poly(cysteine methacrylate). This was shown to give well-defined two-component brushes with predictable brush thickness.

The different reactivity of the POEGMA and PCysMA was exploited to selectively attach a metal chelating group, *N*_α_,*N*_α_-bis(carboxymethyl)-l-lysine, to the surface of the latter brush. This enabled the subsequent attachment of His-tagged proteins, green fluorescent protein and His-tagged proteorhodopsin through nickel chelation. Finally, two-component brushes, where proteorhodopsin was selectively attached to PCysMA, were treated with lipid vesicles to reconstitute proteorhodopsin and prepare a lipid bilayer. Irradiation of the proteorhodopsin with 543 nm light led to a shift in fluorescence emission for the ratiometric dye located within the POEGMA brush layer. This suggests the successful formation of a lipid bilayer, as well as proteorhodopsin-mediated proton pumping across the lipid bilayer.

The ability to prepare two-component polymer brush patterns, where the chemistry of one (or both) brushes can be readily modified for protein conjugation, combined with the use of a ratiometric fluorescent dye label for monitoring the local pH, is expected to enable the design of sophisticated biosensors, as well as facilitating further fundamental studies of membrane proteins.

## Conflicts of interest

There are no conflicts to declare.

## Supplementary Material

Supplementary information
