# Supplementary material for: Fabrication of microstructured binary polymer brush “corrals” with integral pH sensing for studies of proton transport in model membrane systems
Source: Chem Sci. 2018 Jan 15;9(8):2238–51. doi: 10.1039/c7sc04424k (PMC5897877; doi:10.1039/c7sc04424k)
Supplement: Supplementary file 1 [file SC-009-C7SC04424K-s001.pdf]

Electronic Supplementary Material (ESI) for Chemical Science.
This journal is © The Royal Society of Chemistry 2018

Fabrication of Microstructured Binary Polymer Brush "Corrals" with Integral pH Sensing for Studies of Proton Transport in Model Membrane Systems

Jeppe Madsen,[a]† Robert E. Ducker,[a] Omed Al-Jaf,[a‡] Michael L. Cartron,[b] Abdullah M. Alswieleh,[c] Charlie Smith,[a] C. Neil Hunter,[b] Steven P. Armes,[a]* Graham J. Leggett[a]**

[a] *Department of Chemistry, University of Sheffield, Brook Hill, Sheffield S3 7HF, UK.*
[b] *Department of Molecular Biology and Biotechnology, University of Sheffield, Western Bank, Sheffield S10 2TN, UK.*
[c] *King Saud University, Riyadh, Saudi Arabia.*
† Current address: Department of Chemical and Biochemical Engineering, Technical University of Denmark, DK-2800 Kgs. Lyngby, Denmark.
‡ Current address: Department of science, Charmo University, 46023 Chamchamal-Sulaimani, Kurdistan Region, Iraq.
**Corresponding authors. E-mail addresses: JM peterjeppemadsen@googlemail.com; SPA s.p.armes@sheffield.ac.uk; GJL Graham.Leggett@sheffield.ac.uk.*

Experimental Details

Materials

Silicon wafers (reclaimed, p-type, ⟨100⟩) were purchased from Compart Technology (Peterborough, UK), and glass coverslips (22 mm × 60 mm, thickness 1.5) were supplied by Menzel Gläser. Copper(I) bromide (Cu(I)Br, ≥98%), copper(II) bromide (Cu(II)Br, 99.999%), 2,2′-bipyridyl (bpy, ≥99%), N,N-dimethylformamide (DMF, HPLC grade), dimethylphenyl phosphine (DMPP, 99.0%), D-(+)-Gluconic acid δ-lactone (gluconolactone, ≥99%) phosphate buffered saline (PBS), L-cysteine (97%), 3-(acryloyloxy)-2-hydroxypropyl methacrylate (99%), methoxy-capped oligo(ethylene glycol) methacrylate (OEGMA-480, 480 g mol^{-1}), triethylamine (Et_3N ≥99%), α-bromoisobutyryl bromide (BiBB, 98%), (3-aminopropyl)triethoxysilane (APTES, ≥98%), N,N′-Dicyclohexylcarbodiimide (DCC, 99 %), glycerol (99.5 %), 2-isocyanatoethyl methacrylate (98 %), glutaraldehyde (50 % w/w in water), N_α,N_α-Bis(carboxymethyl)-L-lysine hydrate (NTA ≥97%), Nickel(II) Chloride (98%), Phosphoric acid solution (85 % in water), Sodium azide (NaN_3, ≥99 %), tri-*n*-butyl tin hydride ($SnBu_3H$, 97 %) were supplied by Sigma-Aldrich (Poole, UK). Sulfuric acid (s.g. 1.83, >95%), hydrogen peroxide (30% v/v), ammonia solution (s.g. 0.88, 35%), tetrahydrofuran (HPLC grade), ethanol (HPLC grade), toluene (HPLC grade), dichloromethane (HPLC grade), and methanol (HPLC grade) were supplied by Fisher Scientific (Loughborough, UK). 4-(Chloromethyl)phenyltrichlorosilane (CMPTS, 97%) was supplied by Alfa Aesar (Heysham, UK).

Nile Blue A (sulfate) (NB, ≥ 70 %) was obtained from Acros Organics (Geel, Belgium). Sodium hydroxide (NaOH, ≥ 98 %) was supplied by VWR (Lutterworth, UK). Deionized water was prepared using an Elga PURELAB option to 15 MΩ cm. . Buffers for calibration of the pH meter were supplied by ThermoFisher Scientific (Loughborough, UK). Disposable UV grade cuvettes for mounting the glass slides were supplied from Fisher Scientific (Loughborough, UK). 1-palmitoyl-2-oleoyl-*sn*-glycero-3-phosphocholine (>99 %) was purchased from Avanti Lipids (Alabaster, Alabama, US). Texas Red® 1,2-Dihexadecanoyl-*sn*-Glycero-3-Phosphoethanolamine, Triethylammonium Salt (99 %) was supplied by ThermoFisher Scientific (Loughborough, UK). Nile Blue 2-(methacryloyloxy)ethyl

carbamate (NBC) was prepared as described previously.[1]. Cysteine methacrylate was prepared as described previously.[2]

Preparation of proteins

The plasmid containing the pR gene (pBAD-pR) was transformed into E. coli BL21 competent cells. Single colonies of these cells were inoculated into 6 mL of LB medium (plus ampicillin) and grown overnight at 37 °C in the dark with shaking. Next day 600 μL of the overnight culture was used to inoculate 400 mL of LB medium (plus ampicillin) in a conical flask and allowed to grow for 2 h at 37 ºC with shaking. At this point, cells were induced with a final L-arabinose concentration of 0.02 % and 5 mg/mL for all-trans retinal. Cells were allowed to grow for a further 4 h before being spun down and frozen at -20 ºC until further use. The pR protein was purified using a slightly modified version of the protocol used by Gourdon *et al.*[3] Cells from 400 mL growth were thawed at room temperature and mixed with 10 mL of buffer A (20 mM MOPS, pH 7,100 mM NaCl, 1 tablet of Complete EDTA-free protease inhibitor (Roche)) and 0.1 g lysozyme and DNAse. This mixture was left for 30 min at room temperature. The cells were then disrupted in a French pressure cell at 18 000 psi; two cycles of French pressing were used to ensure the majority of cells were disrupted. The resulting mixture was layered onto a discontinuous 15/40% (w/w) sucrose density gradient and centrifuged in a Beckman Ti45 rotor at 27,000 rpm (53,000 g) for 10 h. The membrane band, present just above the 15/40 % interface, was collected using a micropipette then stored at 4 °C overnight or frozen at -20 °C until required. 20 mL of isolated pR containing membranes were thawed. Detergent was then added to a final concentration of 6% w/v solgrade β-OG (Anatrace), and solubilisation was allowed to take place for 1.5 h at 4 ºC with stirring. The sample solution was then loaded onto a 50 mL column packed with chelating sepharose fast flow resin (GE Healthcare) charged with nickel and equilibrated with buffer B (20 mM MOPS, pH 7, 600 mM NaCl, 1% solgrade β-OG and 20 mM imidazole). The column was washed with five column volumes of buffer B, then a gradient of three column volumes was applied ending with 50 mL of 100% buffer C (buffer B + 500 mM imidazole). The fractions containing the pure pR were pooled, concentrated, exchanged with buffer A (+1% solgrade β-OG), then stored at −80 °C until further use.

The gene-encoding GFP was cloned into pET14b (Novagen), and the resulting plasmid was transformed into BL21-competent cells. Single colonies were inoculated into 6 mL of LB plus ampicillin and allowed to grow overnight at 37 °C, then subcultured into a 400 mL LB/ampicillin) in a conical flask. After shaking for 2 h at 37 °C cells were induced with 1 mM IPTG for 4 h, then pelletted and frozen at −20 °C until further use. Cells harvested from a 400 mL culture were resuspended in 10 mL of membrane buffer A (20 mM MOPS, pH 7 100 mM NaCl), and a few grains of DNase I and lysozyme and $MgCl_2$ to 20 mM were added to the suspension and left to incubate at room temperature for 1 h. The cells were then disrupted by two cycles in a French pressure cell at 18 000 psi. The lysate was centrifuged at 15 000 rpm for 25 min, and the supernatant was loaded onto a 50 mL column packed with chelating sepharose fast flow resin (GE Healthcare) charged with nickel and equilibrated with buffer A. The column was washed with five column volumes of buffer A, then a gradient of three column volumes was applied ending with 50 mL of 100% buffer B (buffer A + 500 mM imidazole). The fractions containing the pure GFP were pooled, concentrated, exchanged with buffer A, then stored at −80 °C until further use.

Cleaning of wafers and glass slides

Glass slides and silicon wafers in glass tubes were cleaned thoroughly by immersion in piranha solution (30% hydrogen peroxide, 70% concentrated sulfuric acid) for ca. 30 min. *Warning! If there are excess organic molecules or solvents in glassware, the addition of*

piranha can be explosive. The piranha solution was carefully disposed off by dilution. Substrates were then rinsed by adding deionized water to the container followed by disposal in a total of seven times. The substrates underwent a further clean with a solution of 70% deionized water, 15% hydrogen peroxide, and 15% ammonia solution which was heated and left boiling for 30 min. this was carefully disposed off by dilution and the substrates were washed seven times as before. The substrates were then blown dry using filtered nitrogen to remove excess water and placed in a drying oven at 130 °C for at least 1 h.

Surface amination of wafers and glass slides

Surface amination was conducted using a literature protocol:[4] The clean wafers or microscopy slides were placed in a glass rack in a vacuum desiccator. In a separate vial was added 0.1 mL APTES and this was placed open in the desiccator. The desiccator was evacuated to a pressure of less than 1 mbar and left for 30 min at room temperature. Then the desiccator was opened and the wafers or slides were placed in an oven at 110 °C for 30 min. This procedure was found to give amine-functional surfaces with an R_a roughness value of around 0.10 nm and a sessile drop contact angle of 45-50 °.

Preparation of 2-bromoisobutyryl-based initiator functional surfaces

The reaction of surface amines or alcohols with 2-bromoisobutyryl bromide was carried out at low pressure in a vacuum desiccator: The amino- or alcohol-functional wafers or microscopy slides were placed in a glass rack in a vacuum desiccator. In separate vials were placed 0.1 mL BiBB and 0.3 mL Et_3N respectively and these vials were placed open in the vacuum desiccator. The desiccator was evacuated until Et_3N started boiling (less than 10 mbar) and the reaction was left for at least 12 h and no more than 20 h. The resulting wafers were then washed with ethanol and water and dried under a stream of filtered nitrogen. This procedure was found to give surfaces with an R_a roughness value of 0.11 nm and a sessile drop contact angle of 65-70°. These surfaces were found to be efficient in initiating brush polymerisation and their XPS spectra were indistinguishable from samples made in solution. The kinetics of this gas phase reaction has not been investigated in detail. However, leaving the reaction for only 3 h gave wafers with a relatively lower contact angle, indicating poor coverage of the initiator group and leaving the reaction for more than 20 h gave films that were visually un-even, indicating multilayer formation.

Preparation of 4-chloromethylphenyl trichlorosilane-based initiator functional surfaces

Clean silicon wafers or microscopy slides were immersed in a solution of CMPTS (100 µL, 0.5 mmol) in toluene (30 mL) for 30 min. The wafers were then washed in a mixture of toluene and ethanol (1:1 v:v), ethanol and water. After drying using a stream of filtered nitrogen, the substrates were annealed in a 130 °C oven. These were washed with water prior to use.

Conversion of surface-bound CMPTS to acid groups by irradiation

The chloromethylphenyl groups were oxidized by irradiation with a 244 nm laser (see below) to a total dose of 5 J/cm^2 in air as previously described.[5] These irradiated surfaces had a sessile drop contact angle of around 20 °, which corresponds well with a high surface concentration of carboxylate groups. Patterns were prepared by irradiation through a suitable mask as described below.

Preparation of 2-bromoisobutyryl-based initiator functional surfaces from acid based surfaces

Attachment of 2-bromoisobutyryl groups was carried out in two steps: Initially the acid-functional substrates were immersed in 1 mL of a solution of N,N′-Dicyclohexylcarbodiimide

(0.31 g, 1.5 mmol) in dichloromethane (15 mL). To this mixture was added glycerol (0.010 mL, 0.15 mol) and the mixture was left at 20 °C for 15 h. The hydroxy-functional substrates prepared this way had a sessile drop contact angle of around 40 °. These hydroxy-groups were converted into 2-bromoisobutyrates by reaction with 2-bromoisobutyryl bromide as described above. After esterification, the sessile drop contact angle had increased to 70 °.

Preparation of NBC-labeled and NBC-capped POEGMA-brushes

Fluorescently labelled POEGMA brushes were prepared by modifying a previously published protocol.[6,7] OEGMA-480 (5 g, 10 mmol) was dissolved in water (5 mL), and degassed by passing a stream of nitrogen through the solution for 30 min. Then, bpy (0.07 g, 0.454 mmol), CuBr (0.023 g, 0.16 mmol) and CuBr2 (0.011g, 0.047 mmol) were added to the solution followed by addition of NBC (0.008 g, 0.016 mmol) . The monomer/catalyst mixture was degassed with a stream of nitrogen for 10 min. Initiator functionalized samples were sealed in Schlenk tubes and degassed (vacuum/refill cycles). The monomer/catalyst solution (1 mL) was added to each Schlenk tube from a syringe and the mixture was kept at 30 ± 1 °C. After the desired polymerisation time, the polymerisation was terminated by adding a nitrogen-purged solution (0.5 mL) of NBC (0.0275 g, 0.05 mmol) in ethanol (5 mL). This solution was kept for 15 h at 30 ± 1 °C. The samples were removed, washed with water and ethanol, and dried under a stream of filtered N_2.

Capping of POEGMA brushes with sodium azide

The sodium azide capping was done both in DMF and in Ethanol following a similar procedure.

DMF or ethanol (20 ml) was degassed for 20 minutes and sodium azide added to make up a 0.2 M solution. The samples were placed into reaction vials under nitrogen and the sodium azide solution added (note that the sodium azide is not completely soluble in ethanol, but that it is possible to keep it in suspension with continuous stirring. The solution was added as a suspension using a syringe and needle). After heating the samples overnight at 60 C° they were removed, rinsed with water, ethanol and water again, followed by drying under a stream of nitrogen.

Capping of POEGMA brushes with tin hydride

Capping using tri-*n*-butyl tin hydride was done according to a literature procedure.[8]

Preparation of PCysMA brushes

PCysMA brushes were prepared as described previously.[2] For labelled brushes, NBC was added to the reaction mixture as the stated fraction prior to degassing and the general procedure was followed.

The thickness as a function of time were found not to depend on whether the initiator was based on APTES or irradiated and modified CMPTS (see Figure S3 A), why no further optimisation was carried out.

Preparation of 2-brush structures

2-brush structures were prepared as follows: First, CMPTS wafers were patterned using a 244 nm laser as described above. Then, the resulting carboxylate groups were esterified using DCC and glycerol as described above. This was followed by polymerisation and capping of OEGMA as described above. Then the hydroxyl-groups were reacted with 2-bromoisobutyryl bromide as described above and finally, CysMA was polymerized from the resulting 2-bromoisobutyrate esters as described previously.[2]

Surface-confined reaction between glutaraldehyde and PCysMA brushes

The reaction between the surface amino-groups of PCysMA and glutaraldehyde was carried out after collapsing the PCysMA brush using a poor solvent (tetrahydrofuran). According to literature, This should ensure that only the *surface* groups of the brushes react with the glutaraldehyde.[9,10]

In a typical procedure, a substrate with PCysMA brushes was immersed in THF (1 mL). Then glutaraldehyde solution was added (0.01 mL, 50 % w/w solution, 0.05 mmol) and the mixture was left for 3 h at 20 °C. The substrates were then washed with water and dried under a stream of filtered N_2.

Attachment of N_α,N_α-Bis(carboxymethyl)-L-lysine hydrate and Nickel(II) to aldehyde-functional PCysMA brushes to prepare NTA-functional substrates

The NTA solution was prepared by dissolving N_α,N_α-Bis(carboxymethyl)-L-lysine hydrate (0.01 g, 0.04 mmol) in PBS (10 mL) followed by adjusting the pH to 7.8 with 0.1 M NaOH.

Aldehyde-functional substrates prepared as described above were immersed in the NTA solution (1 mL) for 3 h. The substrates were washed with water and dried under a stream of filtered N_2.

Nickel(II) was attached as previously described.[11] The NTA-functional substrate was incubated with 1 M $NiCl_2$ for 12 h at 20 °C. The substrates were then washed with water and dried under a stream of filtered N_2.

Attachment of His-labeled Proteins to Nickel(II) functional substrates

To the Nickel(II) functional surface was incubated with a solution of His-labeled Protein (Green Fluorescent Protein or proteorhodopsin (0.01 mg/mL) in PBS pH 7.2 for 1 h at 22 °C. The substrate was then washed with PBS pH 7.2 and kept at 4 °C until use.

Formation of lipid bilayers on 2-component brush samples

A literature protocol was followed:[12] To 1-palmitoyl-2-oleoyl-sn-glycero-3-phosphocholine (2.2 mg, 2.9 µmol) was added a solution of 0.15 mM Texas Red® 1,2-Dihexadecanoyl-sn-Glycero-3-Phosphoethanolamine, Triethylammonium Salt in chloroform (0.112 mL). Additional chloroform was added to a total of 5 mL. The solvent was evaporated to dryness using a stream of nitrogen. The resulting solid was then placed in a vacuum desiccator for 2 h.

The lipids were suspended in PBS pH 7.2 through ultrasonication in a bath sonicator for 1 h 15 min to give an opaque suspension.

The suspension was added to the two-component brush samples with or without proteorhodopsin and incubated at 22 °C for 30 minutes. The samples were kept at 4 °C in the lipid suspension until analysis (approximately 16 h) and were washed with PBS and then water (where relevant) immediately before use.

Photopatterning

A Coherent Innova 300C FreD frequency-doubled argon ion laser (Coherent U.K., Ely, UK) with an emission wavelength of 244 nm was used for the UV photodegradation experiments. The energy density was measured prior to each experiment and the dose calculated accordingly. Micro-patterned brushes were obtained by irradiating uniform polymer brush layers on silicon wafer substrates using a copper electron microscopy grid (Agar, Cambridge, UK) as a photomask.

Surface analysis

Sessile contact angle of deionized water drops were measured using a Ramé-Hart model 100-00 contact angle goniometer.

X-ray photoelectron spectroscopy (XPS) was carried out with a Kratos Axis Ultra DLD X-ray photoelectron spectrometer. The instrument had a monochromatic Al Kα X-ray source with an ultrahigh-vacuum environment. Survey and wide scans had acquisition pass energies of 160 and 20 eV, respectively. The XPS data were analyzed using Casa XPS software (UK). All binding energies were calibrated with respect to the C 1s saturated hydrocarbon peak at 285.0 eV.

Secondary mass ion spectrometry (SIMS) was conducted with an Ion time-of-flight SIMS instrument, using an optimized bismuth cluster (Bin) primary ion source incident at 50 keV and with a 500 μm × 500 μm field of view.

Ellipsometry measurements were taken on an M-2000 V ellipsometer (J.A. Woollam Co., Inc.) with a white light source (370.5−998.7 nm) at a 70° incidence angle. The measurements were fitted with a single layer Cauchy model for a polymer brush of n = 1.5 and k = 0 with a silicon substrate (n = 3.875, k = 0.015). Multiple measurements were taken for any given sample, and the brush height quoted was an average of at least three repeat measurements.

AFM studies were carried out in Tapping Mode using a Digital Instruments Multimode 5 Atomic Force Microscope (Veeco, Santa Barbara, USA) with a 'J' scanner (0 – 125 μm). Silicon nanoprobes (Bruker, UK) with nominal force constants of 20-80 Nm^{-1} and tip radii of between 20 and 60 nm were used. Topographic imaging was performed in air.

Confocal Laser Scanning Microscopy with spectral imaging

AN Olympus FV1000 confocal BX61 microscope having spectral imaging capability was used for the acquisition of fluorescence images and for assessing the effect of pH on the fluorescence. Pictures were recorded using Olympus Fluoview FV-ASW software. Post-acquisition analysis was performed using the free image analysis software ImageJ.

Onto patterned samples were added a droplet of phosphate buffer of pH 4, 5, 6, 7, or 8. Onto these samples were placed a cover glass, and the substrate was imaged. In-between buffer changes, the sample was immersed in pure water and the excess water was carefully blotted off using lint-free paper.

The buffers were prepared by adjusting the pH of 0.1 M H_3PO_4 with 1 M NaOH to the recquired pH value. pH was measured using a calibrated pH probe (Hanna Instruments).

Image information was extracted using ImageJ v 1.49 software and the extracted data were treated further in Microsoft Excel.

Fluorescence spectroscopy

Fluorescence spectra of fluorescently labelled brushes on silicon wafers or microscopy slides were obtained using a Hitachi F4500 fluorimeter. The slides were placed in a slide holder prepared in-house mounted in a fluorescence cuvette, where they were positioned at an angle of approximately 100 ° to the excitation beam and the cuvette was filled with a phosphate buffer adjusted to the right pH prior to the measurements. In-between buffer changes, the sample was immersed in pure water and the excess water was carefully blotted off using lint-free paper. Spectra were recorded using the instrument software and exported as 'comma separated value' files for further data treatment in Microsoft Excel.

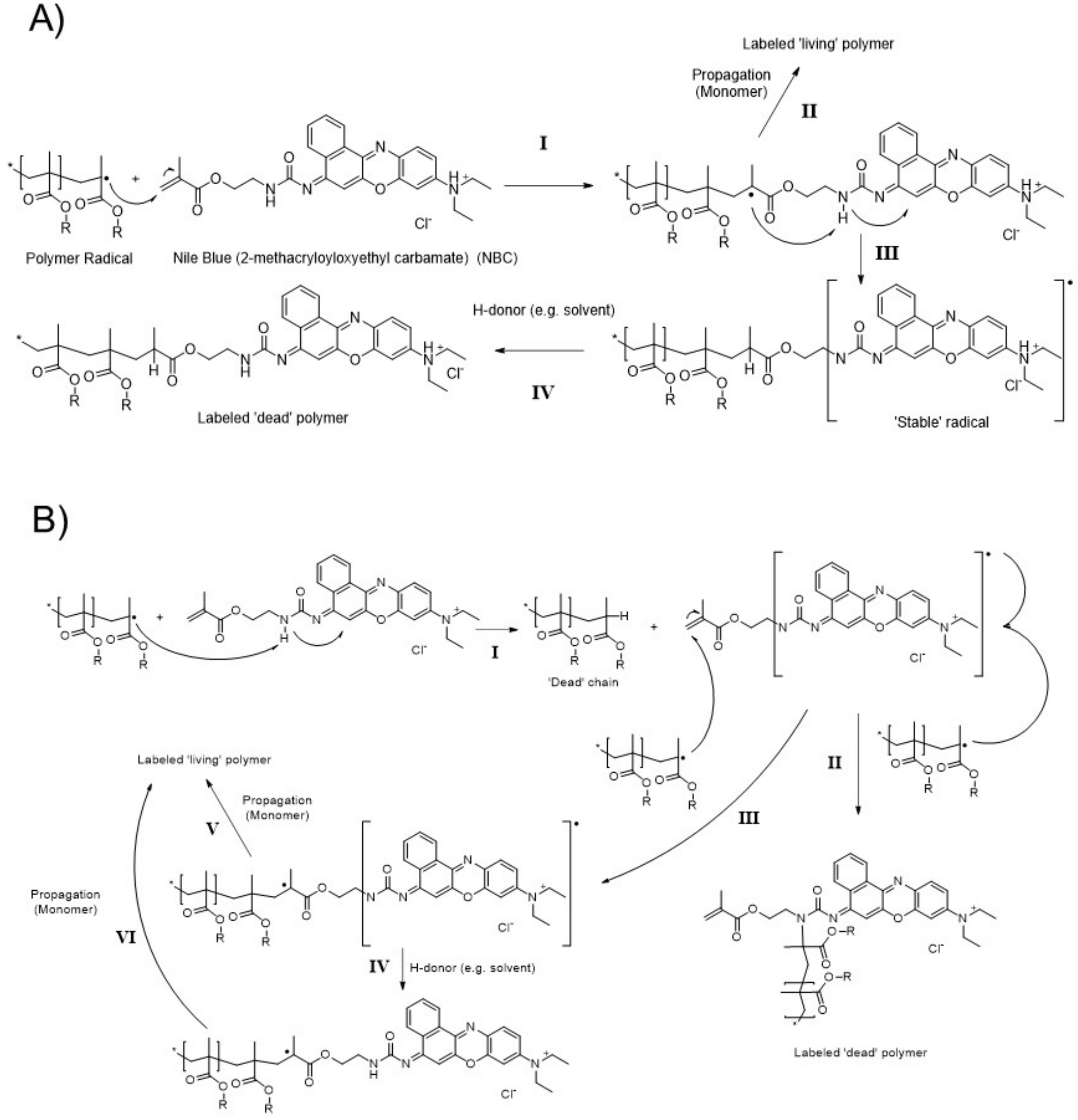

Scheme S1. Possible fates of a radical in the presence of Nile Blue 2-(methacryloyloxy)ethyl carbamate, NBC. A) The propagating radical may react with methacrylic end to give a (macro)-NBC containing radical. This may further propagate to give a labelled 'living' polymer. Alternatively, inter-system crossing is possible to give a non-propagating dye-radical (similar to reactions with aniline [13] and phenoxazines [14]), which eventually will react with a hydrogen donor to give a dye-labelled 'dead' polymer. B) The propagating radical may react with the aromatic system, which will give a 'dead' non-labelled chain and a dye radical. The dye radical can react further with another propagating radical in a radical

recombination reaction to give a labelled ‘dead’ polymer. Alternatively, the radical may react as a monomer through the methacrylate group with another propagating chain, which will give dye-labelled polymers. The latter two examples involve reactions between two radicals to give labelled chains, and as such, these are less likely, at least early in the process where the concentration of dye radicals is very low.

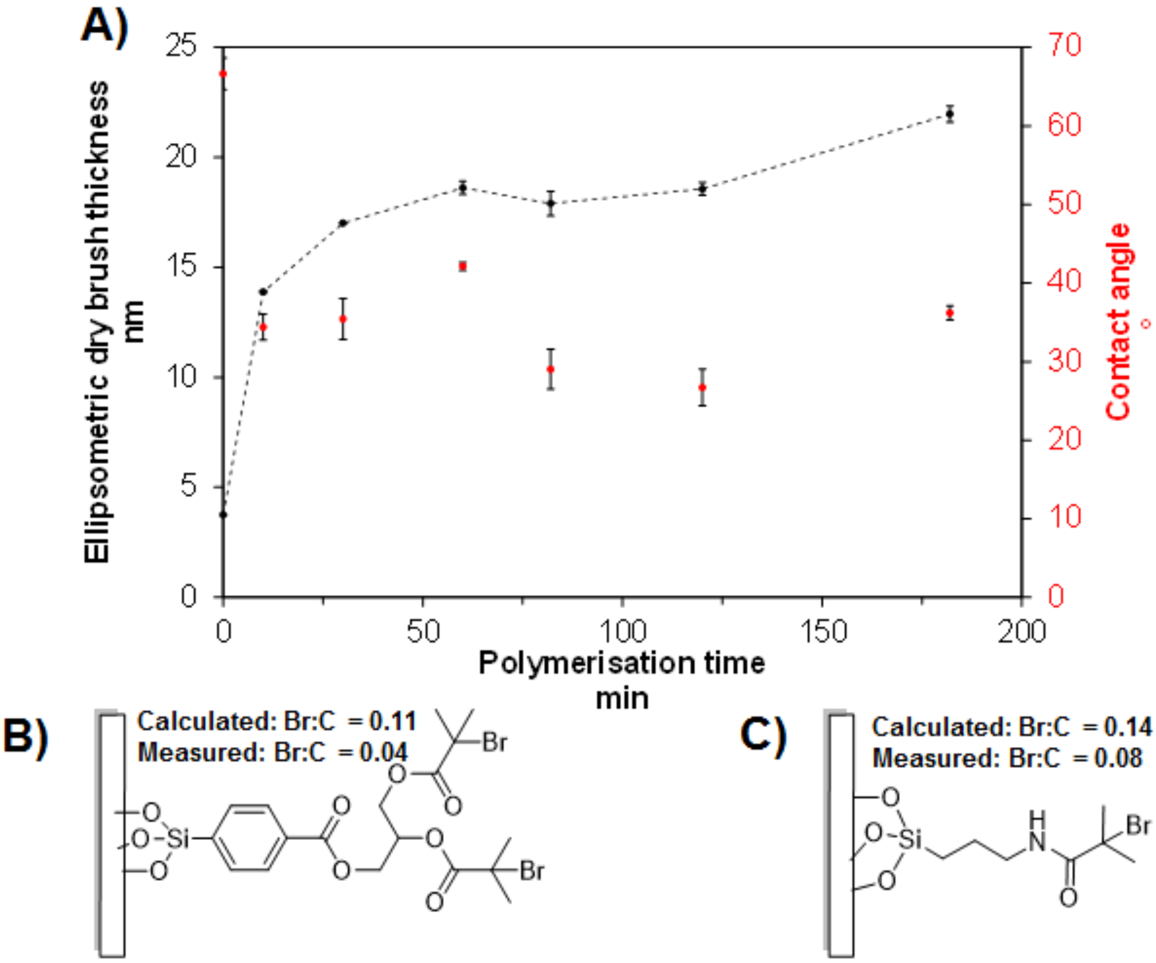

Figure S1. Characterisation of irradiated CMPTS that has been reacted first with glycerol in dichloromethane in the presence of N,N’-dicyclohexylcarbodiimide and then with 2-bromoisobutyryl bromide (BiB) in the presence of triethylamine at reduced pressure. A) Evolution of dry ellipsometric thickness and contact angle on polymerisation of CysMA. Conditions: [CysMA] = 0.88 mol/kg in water. [CysMA]:[bpy]:[Cu(I)Br]:[Cu(II)Br_2] = 140:3:1:0.5, 30 °C. B) Structure of initiator formed from irradiated CMPTS and theoretical and measured ratios between bromine and carbon from wide scan XPS spectra. C) Structure of initiator formed from APTES reacted with BiB (APTES-BiB) for reference, including theoretical and measured ratios between bromine and carbon from wide scan XPS spectra.

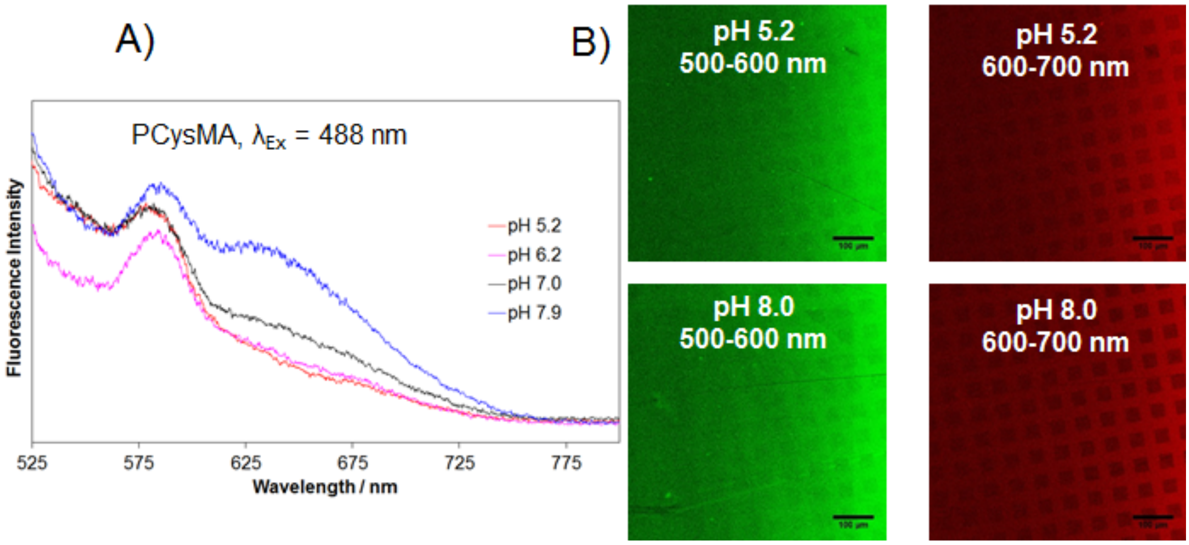

Figure S2. Emission spectra of NBC-labelled PCysMA brushes at pH 5.2 to 7.9. A) Fluorescence emission spectra of a PCysMA-NBC (dry ellipsometric thickness ~10 nm) excited at 488 nm. B) Confocal laser scanning images of PCysMA-NBC (dry ellipsometric thickness ~10 nm) excited at 488 nm at pH 5.2 and pH 8.0 imaged using emission between 500 nm and 600 nm or between 600 nm and 700 nm.

References

(1) Madsen, J.; Canton, I.; Warren, N. J.; Themistou, E.; Blanazs, A.; Ustbas, B.; Tian, X.; Pearson, R.; Battaglia, G.; Lewis, A. L.; Armes, S. P. *J. Am. Chem. Soc.* **2013**, *135*, 14863–14870.

(2) Alswieleh, A. M.; Cheng, N.; Canton, I.; Ustbas, B.; Xue, X.; Ladmiral, V.; Xia, S.; Ducker, R. E.; El Zubir, O.; Cartron, M. L.; Hunter, C. N.; Leggett, G. J.; Armes, S. P. *J. Am. Chem. Soc.* **2014**, *136*, 9404–9413.

(3) Gourdon, P.; Alfredsson, A.; Pedersen, A.; Malmerberg, E.; Nyblom, M.; Widell, M.; Berntsson, R.; Pinhassi, J.; Braiman, M.; Hansson, Ö.; Bonander, N.; Karlsson, G.; Neutze, R. *Protein Expr. Purif.* **2008**, *58*, 103–113.

(4) Willott, J. D.; Murdoch, T. J.; Humphreys, B. A.; Edmondson, S.; Webber, G. B.; Wanless, E. J. *Langmuir* **2014**, *30*, 1827–1836.

(5) Sun, S.; Montague, M.; Critchley, K.; Chen, M. S.; Dressick, W. J.; Evans, S. D.; Leggett, G. J. *Nano Lett.* **2006**, *6*, 29–33.

(6) Brown, A. A.; Khan, N. S.; Steinbock, L.; Huck, W. T. S. *Eur. Polym. J.* **2005**, *41*, 1757–1765.

(7) Alswieleh, A. M. M. *Ph. D. Thesis Univ. Sheff.* **2014**, July.

(8) Ionov, L.; Minko, S. *ACS Appl. Mater. Interfaces* **2012**, *4*, 483–489.

(9) Cheng, N.; Bao, P.; Evans, S. D.; Leggett, G. J.; Armes, S. P. *Macromolecules* **2015**, *48*, 3095–3103.

(10) Alswieleh, A. M.; Cheng, N.; Leggett, G. J.; Armes, S. P. *Langmuir* **2014**, *30*, 1391–1400.

(11) Wang, W.; Wang, D. I. C.; Li, Z. *Chem. Commun.* **2011**, *47*, 8115–8117.

(12) Smith, E. A.; Coym, J. W.; Cowell, S. M.; Tokimoto, T.; Hruby, V. J.; Yamamura, H.

I.; Wirth, M. J. *Langmuir* **2005**, *21*, 9644–9650.

(13) Wei, Y.; Hariharan, R.; Patel, S. a. *Macromolecules* **1990**, *23*, 758–764.

(14) Lucarini, M.; Pedrielli, P.; Pedulli, G. F.; Valgimigli, L.; Gigmes, D.; Tordo, P. *J. Am. Chem. Soc.* **1999**, *121*, 11546–11553.
